# Research on the threshold of the supply and demand of ecosystem services

**DOI:** 10.1371/journal.pone.0339122

**Published:** 2026-02-02

**Authors:** Huangwei Deng, Zhenliang Liao, Xuefei Zhou

**Affiliations:** 1 College of Environmental Science and Engineering, Tongji University, Shanghai, China; 2 United Nations Environment Programme-Tongji Institute of Environment for Sustainable Development, Tongji University, Shanghai, China; 3 State Key Laboratory of Pollution Control and Resources Reuse, College of Environmental Science and Engineering, Tongji University, Shanghai, China; Endocrinology and Metabolism Population Sciences Institute, Tehran University of Medical Sciences, IRAN, ISLAMIC REPUBLIC OF

## Abstract

The ecological threshold has not yet formed a unified definition, and there is no definition for “the threshold of the supply and demand of ecosystem services (*Tr*_*SD*_)”, leading to no limitation of the negative impact of production and life behavior on the supply and demand of ecosystem services. This study defined and set *Tr*_*SD*_, and took Urumqi as an example to carry out a case study. Firstly, the concept of *Tr*_*SD*_ was elaborated referred to multiple definitions of the ecological threshold based on “the difference between the supply and demand of ecosystem services (*ES*_*r*_)”. Then, the geographical simulation and optimization system- future land use simulation (GeoSOS-FLUS) software was used to simulate future land use. After that, the Land Use and Land Cover (LULC) matrix model was applied to calculate *ES*_*r*_. Finally, the *Tr*_*SD*_ was determined via the inflection point analysis of *ES*_*r*_. This study concludes that the proposed *Tr*_*SD*_ and its systematic calculation method are innovative and rational. The results can be used for ecosystem service management and ecological valuation, which helps the sustainability progress of the global.

## 1 Introduction

Ecosystem service was first proposed by Holden and Ehrlich in 1974 and defined by the Millennium Ecosystem Assessment as the benefits provided by ecosystems to humans [1]. In the process of social development, human demand for economy and ecosystem services is increasing. However, the supply of ecosystem services is limited, which means there may be a mismatch between the supply and demand of ecosystem services.

Ecosystem resilience is the capacity of an ecosystem to maintain its key functions and reorganize following disturbance. When the resilience of an ecosystem is sufficiently degraded due to disturbances, the system will transition from an ideal state to a high-risk state, leading to the emergence of ecological thresholds [2]. In the context of ecological thresholds, even minor changes in disturbance can cause shifts in ecosystem states [3]. In nature, ecological thresholds primarily exist in two forms: “points” and “zones.” Simply put, a “point” threshold describes an immediate condition, such as a species on the brink of extinction, while a “zone” threshold depicts the transformation process of ecosystem states [4]. Due to stresses from both internal and external factors, ecosystems undergo changes in structure and function. Once these stresses exceed certain thresholds, significant changes in ecosystem states occur [5]. Therefore, ecological thresholds are particularly important for environmental management and sustainable development.

Ecological threshold describes the process by which quantitative change leads to a qualitative change in ecosystems, it is an important indicator of urban planning. The Threshold Alliance listed nearly 50 different definitions of “ecological thresholds” based on studies such as the state of different ecosystems [6]. For example (shown in Table 1), the carrying capacity of the ecosystem mainly emphasized the stress of all biological and human activities in the area carried by the ecosystem [7]. The planetary boundary sets the safety boundary of key biophysical processes for the earth system [8,9]. Tang et al. [10] consider ecological thresholds as the critical values that cause divergence or abrupt changes in ecosystem processes or states. The abrupt changes in ecosystems stem from the accumulation of changes in resource and environmental factors during the evolution of ecosystems or the occurrence of extreme events, manifesting as a turning point in the changes of ecosystem structure and function [11]. Overall, current research lacks an analysis of the concept of “threshold” and its setting from the perspective of ecosystem service supply and demand, failing to provide guidance for controlling the balance between ecosystem service supply and demand.

**Table 1:** Different meanings of ecological thresholds.

Name	Meaning
The carrying capacity of the ecosystem	Emphasized the stress of all biological and human activities in the area carried by the ecosystem
The planetary boundary (environmental damage threshold)	The safety boundary of key biophysical processes for the earth system
Ecological thresholds	The critical values that cause divergence or abrupt changes in ecosystem processes or states
The threshold of the supply and demand of ecosystem services	The safety limit of the difference between the supply and demand of ecosystem services

To address the potential ecological risks resulting from the lack of threshold settings for the supply-demand imbalance of ecosystem services, this study propose the concept of the threshlod of the supply and demand of ecosystem services (*Tr*_*SD*_) (All abbreviations are listed in Table 2). This study tries to define and set *Tr*_*SD*_ based on “the difference between the supply and demand of ecosystem services (*ES*_*r*_)” to maintain the continuous surplus of the supply and demand of ecosystem services and promote eco-friendly development. To set *Tr*_*SD*_, the changes in *ES*_*r*_ should be identified, and future land use should be predicted first for *ES*_*r*_ calculation.

**Table 2:** Main abbreviations.

Abbreviation	Interpretation
*Tr*_*SD*_	the threshold of the supply and demand of ecosystem services
*ES*_*r*_	the difference between the supply and demand of ecosystem services
GeoSOS-FLUS	the geographical simulation and optimization system- future land use simulation
LULC matrix	Land Use and Land Cover matrix
*ES*_*s*_	the supply of ecosystem service
*ES*_*d*_	the demand for ecosystem services
*R*_*SD*_	the matching degree of ecosystem services supply and demand
*C*_*SD*_	the coordination of ecosystem services supply and demand
*ES*_*r0*_	the value of *ES*_*r*_ at the tipping point of the difference between the supply and demand of ecosystem services
BS	basic scenario
E_n_F	economy-first scenario
E_s_F	ecology-first scenario
SD	sustainable development scenario
*i*	the classification of ecosystem services
*j*	LULC type
*k*_*js*_	the intensities of the supply of ecosystem services corresponding to the specific LULC type
*S*_*j*_	the area of the specific LULC type
*k*_*jd*_	the intensities of the demand for ecosystem services corresponding to the specific LULC type
*f(x)*	function of *ES*_*r*_
(*x*_*0*_, *f(x*_*0*_))	the inflection point

Regarding future land use and land cover prediction, cellular automata-Markov (CA-Markov), future land use simulation (FLUS) [12], geographical simulation and optimization system-future land use simulation (GeoSOS-FLUS), and conversion of land use and its effects at small region extent (CLUE-S) [13] are used for future land use prediction. Among them, GeoSOS-FLUS integrates CA-Markov and FLUS models, which can predict land use data (top-down quantitative simulation) and simulate the spatial distribution of land use (bottom-up spatial simulation) [14,15]. What’s more, it predicts future land use based on several driving factors, which can effectively deal with the common uncertainty of human activities and nature [16].

Regarding the evaluation of the supply and demand of ecosystem services, there are several methods proposed in research works, such as the land use and land cover (LULC) matrix model, integrated valuation of ecosystem services and trade-offs (InVEST), ecological footprint (EF), ecosystem services provision Index (ESPI), and land development index (LDI). Among them, the LULC matrix model can calculate the supply and demand of ecosystem services simultaneously, which only requires data on land use and the intensities of ecosystem services. The LULC matrix model establishes an ecosystem services’ supply matrix and an ecosystem services’ demand matrix to quantify the supply and the demand of ecosystem services, respectively. [17,18].

At present, the determination method of ecological thresholds mainly contains the experimental observation [2,19–21], the numerical model simulation [4,22], and the statistical analysis [23]. The inflection point analysis is a kind of statistical analysis tool, and is usually used for data analysis in the field of finance, energy consumption, internet business, etc. It is easy to operate and has a great application possibility in the field of ecological researches.

**Fig 1:**
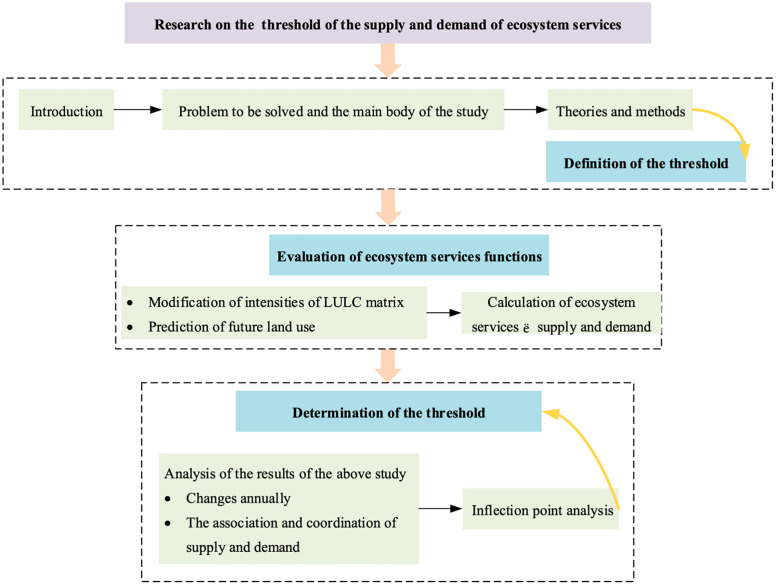
Research Framework.

According to the above, this study mainly intends to define *Tr*_*SD*_ based on the supply and demand of ecosystem services and propose a systematic method of *Tr*_*SD*_ determination based on *ES*_*r*_, giving suggestions for land planning. Regarding *Tr*_*SD*_ determination, this study works in three steps: a) Obtain and predict land use/ land change data of the study area; b) Modify the intensities of LULC matrix and calculate *ES*_*r*_; c) Set *Tr*_*SD*_ via the inflection point analysis of *ES*_*r*_. The framework of this study is shown in Fig 1.

Display the framework of the study and the structure of the article.

In this study, Section 2 contains the definition of *Tr*_*SD*_, and the methods of determining *Tr*_*SD*_. Section 3 presents the results of the case study. Section 4 makes discussions on the definition, results, and methods. Section 5 is the conclusions.

## 2 Case and methods

This section contains four parts. Section 2.1 and Section 2.2 introduces the basic information of the case city and data sources, respectively. Section 2.3 expresses the definition of *Tr*_*SD*_. Section 2.4 introduces the methods for *Tr*_*SD*_ determination, including the GeoSOS-FLUS model (future land use prediction), LULC matrix model with modified ecosystem services’ intensities (*ES*_*r*_ evaluation), and inflection point analysis of *ES*_*r*_.

### 2.1 Study area

**Fig 2:**
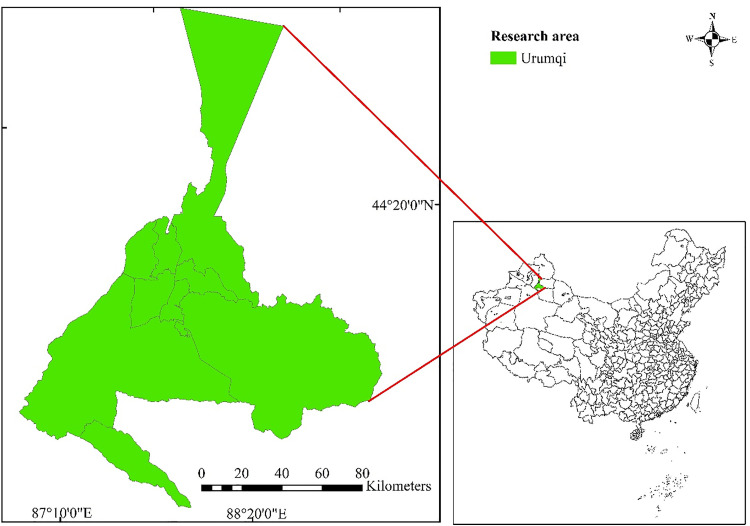
Case area.

Urumqi (86°37′33″-88°58′24″E, 42°45′32″-44°08′00″N) is located in northwest China and is the capital of Xinjiang Uygur Autonomous Region, shown in Fig 2. Urumqi is the central area of the core area of the Silk Road Economic Belt, surrounded by mountains on three sides, with a variety of land cover forms and has unique energy resource advantages as well as rich animal and plant resources. Table 3 displays the situation of land use in Urumqi in the past few years. However, Urumqi belongs to an arid area with little precipitation and faces ecological security threats such as ecological sensitivity and fragility due to historical factors. In recent years, the economy and urbanization process of Urumqi has developed rapidly. Strengthening ecological environmental protection and optimizing construction while striving to develop a social economy is the top priority of Urumqi’s current development. It has been emphasized that the development of Urumqi shall adhere to the strategy of sustainable development, continuously improve the ecological environment, and comprehensively improve the quality of the ecosystem. Therefore, from the perspective of development goals and ecological protection, this study chose Urumqi City as the case area.

**Table 3:** Historical land use data of Urumqi (Unit: square kilometers).

Year	1990	1995	2000	2005	2010	2015	2020
Farmland	1014.1137	965.4651	898.3278	938.6532	902.0043	762.6168	671.9607
Forest	457.8345	480.3363	494.3088	519.345	539.8011	554.2569	567.7992
Shrub	0.0342	0.0675	0.0054	0.0054	0.0045	0.0045	0.0792
Grassland	7306.6644	7368.8661	7457.0211	7176.8961	7024.5261	7036.6941	6842.4147
Water area	65.1339	72.6219	83.9439	94.1877	102.6702	92.2356	95.5413
Snow and ice	176.6592	211.9068	189.2358	178.5447	190.1142	217.9206	204.5385
Bare land	5052.9771	4917.879	4843.5417	5019.4647	5100.1209	5147.1288	5381.7273
Impervious surface	133.8003	190.08	240.8427	280.1295	347.9841	396.3591	443.1519
Wetland	0.0117	0.0063	0.0018	0.0027	0.0036	0.0126	0.0162

The simple map of the case area.

### 2.2 Data sources

The thesis collected remote sensing images of land monitoring, digital elevation model, and another necessary data from different resources. The details were shown in Table 4. The availiblity of these data resources were explained in the file “Supporting information”.

**Table 4:** Data resources.

Data	Resources
Remote sensing images of land monitoring	The years 1990, 1995, 2000, 2005, 2010, 2015, and 2020 [Jie Yang, & Xin Huang. (2022). The 30 m annual land cover datasets and its dynamics in China from 1990 to 2021 (1.0.0).]. The precision of remote sensing is 30m.
Digital elevation model (DEM)	RESDC (https://www.resdc.cn/)
Grids of people, annual precipitation, annual temperature, and gross domestic product (GDP)	
Grids of road nets and water	National Catalogue service for geographic information (https://www.webmap.cn/commres.do?method=result25W)
Other social and economic data	National Data (https://data.stats.gov.cn/)
	The statistical yearbook for the corresponding year

### 2.3 Definition of the threshold of the supply and demand of the ecosystem services

The supply and demand of ecosystem services change with human actives and ecosystem activities. According to the general concept of threshold, ***Tr***_***SD***_ **refers to the state in which the difference between the supply and demand of ecosystem services arrives at a tipping point.** Within *Tr*_*SD*_, the ecosystem provides sustainable ecosystem services with no significant jump in *ES*_*r*_, the supply of ecosystem services can maintain stable demand for ecosystem services, and the supply of ecosystem services is in good coordination with the demand for ecosystem services.

The *ES*_*r*_ can be calculated by the following equation:


$${ES}_{r}={ES}_{s}-{ES}_{d}$$ (2.1)

Among them, *ES*_*s*_ represents the supply of ecosystem service, *ES*_*d*_ represents the demand for ecosystem services. The calculation of *ES*_*s*_ and *ES*_*d*_ are introduced in Section 2.4.2. A positive value of *ES*_*r*_ indicates that supply exceeds demand, which means a surplus of ecosystem services, while a negative value indicates that supply is less than demand, which means an ecosystem services deficit.

Referring to Sun’s [24], Guan’s [25], and Chen’s [26] research works on the association and coordination of ecosystem services supply and demand, this study listed the calculation methods of the ratio and coordination of ecosystem services supply and demand.

*R*_*SD*_ refers to the matching degree of ecosystem services supply and demand. It can be calculated by:


$$R_{SD}=\frac{{ES}_{s}}{{ES}_{d}}$$ (2.2)

*R*_*SD*_ > 1 means the ecosystem service supply can maintain stable demand for ecosystem services, and the relationship between the supply and demand of ecosystem services is stable and harmonious. *R*_*SD*_ = 1 indicates that the supply and demand of ecosystem services are saturated. *R*_*SD*_ < 1 means that the supply of ecosystem services cannot maintain stable demand for ecosystem services, resulting in a conflict between supply and demand [27].

*C*_*SD*_ means the coordination of ecosystem services supply and demand. It can be calculated by:


$$C_{SD}=\sqrt{\left[\frac{{ES}_{s}\times {ES}_{d}}{{\left(\frac{{ES}_{s}+{ES}_{d}}{\text{2}}\right)}^{\text{2}}}\right]}$$ (2.3)

To ensure the coordination of the supply and demand of ecosystem services, the value of *C*_*SD*_ shall be larger than 0.5. *C*_*SD*_ > 0.8 means the state of the supply and demand of ecosystem services is well [28].

Within *Tr*_*SD*_, it should satisfy the equation:


$${Tr}_{SD}={ES}_{r\text{0}},\left\{ \;\begin{array}{c} {ES}_{r\text{0}}>\text{0};\\ {C_{SD}\geq \text{0}.\text{5},\;R}_{SD}\geq \text{1}. \end{array}\right.\;$$ (2.4)

Among them, *ES*_*r0*_ is the value of *ES*_*r*_ at the tipping point of the difference between the supply and demand of ecosystem services.

### 2.4 Methods

According to Section 2.3, the changes in *ES*_*r*_ shall be identified to determine *Tr*_*SD*_. Thus, the calculation of *ES*_*r*_ shall be conducted. Regarding future *ES*_*r*_ calculation, future land use shall be predicted first. This study proposed a *Tr*_*SD*_ determination method based on its definition and the supply and demand of ecosystem services, including three steps:

Step 1: Build different future development scenarios, and predict future land use change via GeoSOS-FLUS.

Step 2: Calculate the supply and demand of ecosystem services via the modified LULC matrix model.

Step 3: Make an inflection point analysis on *ES*_*r*_, and determine *Tr*_*SD*_ according to the results of inflection point analysis.

#### 2.4.1 Future land use prediction.

**Fig 3:**
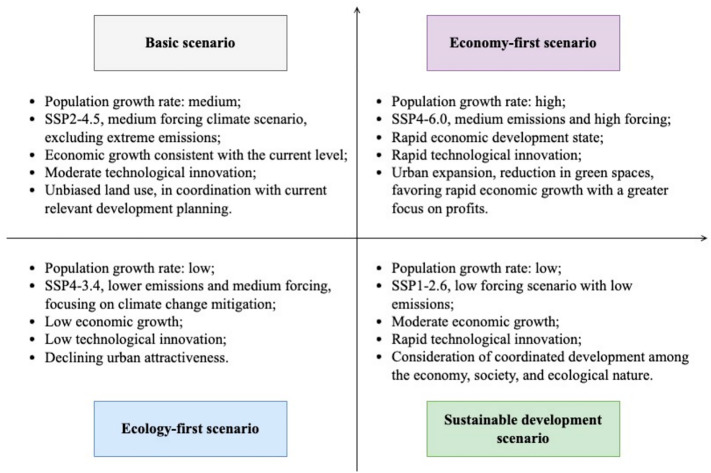
Future development scenarios.

The socio-economic development changes, the development of industry and agriculture, as well as urbanization processes drive changes in land use. This study established four development scenarios for future land use [13,15,16,29]: basic scenario (BS), economy-first scenario (E_n_F), ecology-first scenario (E_s_F), and sustainable development scenario (SD), as Fig 3 shows.

Explain four development scenarios for future land use: basic scenario (BS), economy-first scenario (E_n_F), ecology-first scenario (E_s_F), and sustainable development scenario (SD).

**Fig 4:**
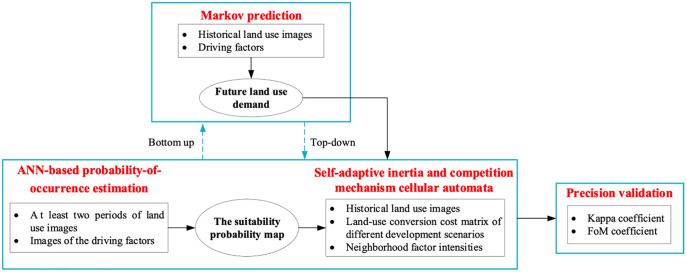
GeoSOS-FLUS modules.

This study took use of the GeoSOS-FLUS model to predict future land use in different scenarios. The model contains four modules, shown in Fig 4 [13,16]. Appendix A shows the details of the modules of the GeoSOS-FLUS model S2 File.

The modules and their functions of the GeoSOS-FLUS model.

**Fig 5:**
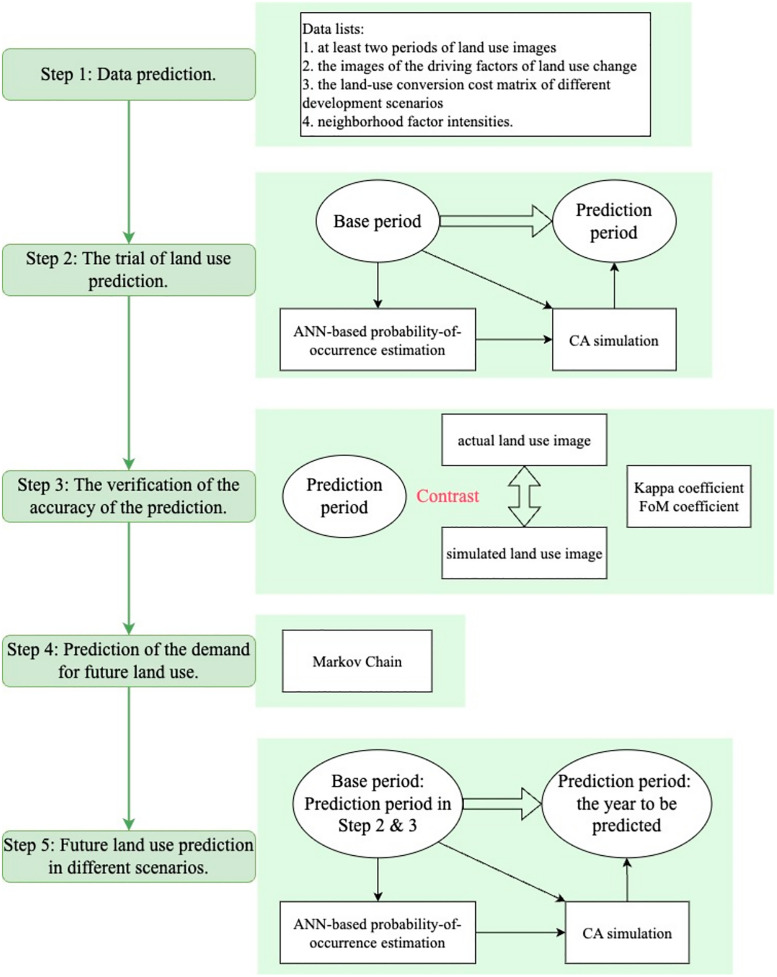
Steps of GeoSOS-FLUS’s operation.

The deficit operation of GeoSOS-FLUS model was presented by the following five steps, shown in Fig 5. The details of the deficit operation to predict future lande use can be seen in Appendix B S2 File.

The five steps of the GeoSOS-FLUS model for predicting future land use.

#### 2.4.2 Quantification of ecosystem services.

a) **Intensities’ modification of LULC matrix**

In Burkhard’s studies [17,18,30], the land use types were similar to Coordination of Information on the Environment (CORINE), which was different from this study. The intensities of the supply and demand of ecosystem services in the LULC matrix are related to land use. To reduce the degree of inaccurate results caused by the difference, the study should modify the intensities of ecosystem services for the LULC matrix model.

Based on land use types and ecosystem service function types in this study, the intensities of the LULC matrix were modified by comparing and referring to the relevant articles [17,18,24,25,30,31]. The detailed processes are as follows, the same as that in Deng et al’s research [27].

Step 1: Compare the differences in the chosen ecosystem services. Firstly, ascertain the content of provisioning services, regulating services, and cultural services in this study, respectively. Then, compare the contents of this study with that of Wu’s [32], Burkhard’s [17,18,30], Sun’s [24], and Tao’s [31] research works.

Step 2: Analyze LULC types, and establish the LULC matrix model. After the implementation of step 1, compare the LULC types of different land cover systems to collect the intensities of ecosystem services.

Step 3: Modify the intensities of ecosystem services. Based on step 2, take the average value of the similarity or same intensities shown in Wu’s [32], Burkhard’s [17,18,30], Sun’s [24], and Tao’s [31] researches. The mean values of the calculation are the intensities of the corresponding ecosystem services supply and demand in the LULC matrix. The supply-demand intensities of ecosystem services were calculated by subtracting the intensities of the supply matrix and the intensities of the demand matrix. The results are shown in Table 5.

**Table 5:** The intensities of the supply matrix [1], demand matrix [2], and the difference between the supply matrix and demand matrix [3] of ecosystem services.

	Provisioning services			Regulating services			Cultural services			Total 1	Total 2	Total 3
	1	2	3	1	2	3	1	2	3			
Farmland	9.2	5.3	3.9	7.3	17	−9.7	5.5	0	5.5	**22**	**22.3**	**−0.3**
Forest	4.5	3	1.5	22.6	0	22.6	12.3	0	12.3	**39.4**	**3**	**36.4**
Shrub	5	0	5	17.5	0	17.5	5	0	5	**27.5**	**0**	**27.5**
Grassland	4.5	4	0.5	10.5	4	6.5	7.5	0	7.5	**22.5**	**8**	**14.5**
Water area	5	3	2	9.5	0	9.5	14	0	14	**28.5**	**3**	**25.5**
Snow and ice	5	0	5	6	0	6	10	0	10	**21**	**0**	**21**
Bare land	0	0	0	3	0	3	6	0	6	**9**	**0**	**9**
Impervious surface	2	11.5	−9.5	4.5	16.3	−11.8	2	5	−3	**8.5**	**32.8**	**−24.3**
Wetland	2.5	3	−0.5	6.6	0	6.6	9.4	0	9.4	**18.5**	**3**	**15.5**

b) **LULC Matrix calculation**

To determine the threshold of the supply and demand of ecosystem services, the first is to assess *ES*_*r*_. LULC matrix model makes use of local LULC, with no need for more data, which is more available for this study. The detailed calculations of the supply and demand of ecosystem services are as follows.

The supply of ecosystem services:


$${ES}_{s}=\sum_{i=\text{1}}^{\text{3}}\sum_{j=\text{1}}^{\text{9}}S_{j}\times k_{js}$$ (2.5)

Among them, *i* represents the classification of ecosystem services, *i = 1,2,3,...*, that is, provisioning services, regulating services, and cultural services.*j* represents LULC type, *j = 1,2,...,9*. $S_{j}$ is the area of the specific LULC type, km^2^; *k*_*js*_ represents the intensities of the supply of ecosystem services corresponding to the specific LULC type.

The demand for ecosystem services:


$${ES}_{d}=\sum_{i=\text{1}}^{\text{3}}\sum_{j=\text{1}}^{\text{9}}S_{j}\times k_{jd}$$ (2.6)

Among them, *i* represents the classification of ecosystem services,*i = 1,2,3,...*, that is, provisioning services, regulating services, and cultural services. *j* represents LULC type, *j = 1,2,...,9*. *S*_*j*_ is the area of the specific LULC type, km^2^; *k*_*jd*_ represents the intensities of the demand for ecosystem services corresponding to the specific LULC type.

#### 2.4.3 Inflection point analysis.

At present, statistical analysis and simulation models are common methods for determining thresholds [5,7]. In general, the determination of threshold mainly adopts mean analysis, inflection point analysis, two-eight rule, quartile analysis, and standard deviation confirmation methods. *Tr*_*SD*_ in this study is a macroscopic demonstration of *ES*_*r*_. According to the definition of *Tr*_*SD*_ in Section 2.3, its goal is to ensure the surplus of the supply and demand of ecosystem services. To determine *Tr*_*SD*_ is to find the tipping point of *ES*_*r*_. In the absence of a large amount of field data, the threshold can be set based on the change in the supply and demand of ecosystem services. This study intends to use inflection point analysis to analyze and confirm *Tr*_*SD*_.

The inflection point is the concave and convex dividing point of a continuous and smooth function *f(x)* curve. Regarding the inflection point (*x*_*0*_, *f(x*_*0*_*)*), for any δ (δ > 0), it shall satisfy the equation:


$${ES}_{s}=\sum_{i=\text{1}}^{\text{3}}\sum_{j=\text{1}}^{\text{9}}S_{j}\times k_{js}$$ (2.7)

Among them, *x* represents the year, *f(x)* is *ES*_*r*_. *Tr*_*SD*_ can be regarded as the *f(x*_*0*_*)*. According to the definition of *Tr*_*SD*_, the tipping point of *ES*_*r*_ is regarded as *Tr*_*SD*_, it is used to ensure the surplus of the supply and demand of ecosystem services. For the function *f(x)* with more than one inflection point, in accordance with the principle of the primacy of ecological protection, the minimum *f(x*_*0*_*)* which satisfies the requirements listed in equation (2.4) is regarded as *Tr*_*SD*_.

## 3 Results

This study predicted future land use in Urumqi via the GeoSOS-FLUS model introduced in Section 2.4.1, calculated the supply and demand of ecosystem services via the modified LULC matrix model introduced in Section 2.4.2, and determined *Tr*_*SD*_ via inflection point analysis of *ES*_*r*_ introduced in Section 2.4.3. The results are presented as follows.

### 3.1 Future land use

According to the method introduced in Section 2.4.1, this study predicted future land use in Urumqi. This study chose random sampling in ANN-based probability-of-occurrence estimation, the sampling rate was 20/1000 and had 12 hidden layers. The demands for future land use were predicted via the Markov chain in Section 2.4.1, and the results were shown in Table 6.

**Table 6:** The demands of future land use (Unit: Pixels).

Future scenario	Year	Farmland	Forest	Shrub	Grassland	Water area	Snow and ice	Bare land	Impervious surface	Wetland
BS	2030	846347	588211	0	7408021	98950	243058	6232590	368632	1
	2060	668800	571277	0	6868337	81006	288788	6964434	343168	0
	2080	578816	560259	0	6543496	71592	317757	7386713	327177	0
	2100	506010	549453	0	6244669	63815	345422	7764509	311931	0
E_n_F	2030	994846	660170	5	7618050	108247	242994	5564622	596848	28
	2060	982883	737839	5	7358071	100785	288517	5414797	902869	45
	2080	974412	781960	5	7196970	96546	317588	5317162	1101118	50
	2100	965615	820636	5	7044617	92810	345730	5221287	1295058	52
E_s_F	2030	996063	660925	5	7837943	107248	243065	5564836	375696	28
	2060	987424	745007	5	7889870	98971	289261	5415318	359909	45
	2080	982076	796436	5	7925827	94554	319189	5317878	349795	50
	2100	977058	844504	5	7962597	90874	348532	5222191	339996	53
SD	2030	1000347	662118	5	7861308	110873	212393	5565711	373026	28
	2060	997835	756448	5	7940085	106330	214041	5417431	353587	47
	2080	996341	819848	5	7988979	103470	215086	5320768	341261	52
	2100	994976	883630	5	8035081	100737	216089	5225821	329415	56

Referring to Li’s [33], Liu’s [16], and Chen’s [34] research works, the cost matrixs of future scenarios were set and shown in Table 7. Regarding the weight of the neighborhood in self-adaptive inertia and competition mechanism CA, they were set on the condition that the result of Kappa ranged from 0.8 to 1 [35], and the result of FoM ranged from 0.01 to 0.5 [36]. The results were shown in Table 8. The Kappa and FoM were 0.833774 and 0.102655, separately, which meant the simulation results in this study were credible.

**Table 7:** The cost matrix of future scenarios.

SD	1	2	3	4	5	6	7	8	9	E_S_F	1	2	3	4	5	6	7	8	9	E_n_F	1	2	3	4	5	6	7	8	9
1	1	1	1	1	1	0	0	0	1	1	1	1	1	1	1	1	0	0	1	1	1	0	1	1	1	1	0	0	1
2	0	1	0	0	0	0	0	0	0	2	1	1	1	1	1	1	0	0	1	2	1	1	1	1	1	0	0	1	1
3	0	1	1	0	0	0	0	0	0	3	1	1	1	1	1	1	0	0	1	3	1	1	1	1	1	0	0	1	1
4	1	1	1	1	1	0	0	0	1	4	1	1	1	1	1	1	0	0	1	4	1	1	1	1	1	1	0	1	1
5	1	1	1	1	1	0	0	0	0	5	1	1	1	1	1	1	0	0	1	5	1	0	0	0	1	1	0	1	1
6	1	1	1	1	1	1	0	0	1	6	1	1	1	1	1	1	0	0	1	6	1	0	0	0	1	1	0	1	1
7	1	1	1	1	1	1	1	1	1	7	1	1	1	1	1	1	1	1	1	7	1	1	1	1	1	1	1	1	1
8	1	1	1	1	1	1	1	1	1	8	0	0	0	1	0	0	0	1	1	8	0	0	0	0	0	0	0	1	0
9	1	1	1	1	1	0	0	0	1	9	1	1	1	1	1	1	0	0	1	9	1	0	0	0	1	1	0	1	1

**Table 8:** The weight of the neighborhood.

Land use type	Farmland	Forest	Shrub	Grassland	Water area	Snow and ice	Bare land	Impervious surface	Wetland
Weight	0.3	0.6	0.6	0.5	0.4	0.2	0.1	1	0.4

To identify long-term variation, this study predicted future land use in 2030, 2060, and 2100 via self-adaptive inertia and competition mechanism CA in GeoSOS-FLUS. Appendix C (including future land use of the four scenarios) displays the spatial distributions of future land use of Urumqi S2 File.

The land use data were calculated via ArcGIS 10.8, and the results were shown in Table 9. Under BS, the area of farmland will be significantly reduced, and the impervious surface will be maintained at a relatively stable level after increasing to a certain extent. Under E_n_F, urban expansion will continue to increase, and the proportion of impervious surfaces will continue to increase. Under E_s_F, the proportion of forest and grassland will increase, and some bare land and impervious surface will turn into farmland and green land. Under SD, the area of forest, shrub, grassland, water area and wetland will increase significantly, mainly from the transformation of impervious surfaces and bare land.

**Table 9:** The results of future land use (Unit: square kilometers).

Future scenario	Year	Farmland	Forest	Shrub	Grassland	Water area	Snow and ice	Bare land	Impervious surface	Wetland
BS	2030	761.715	571.176	0.0657	6667.219	89.055	207.0432	5523.207	387.7389	0.009
	2060	601.92	569.3202	0.0702	6181.502	72.9054	259.9092	6094.008	427.5846	0.009
	2080	531.684	568.2528	0.0639	5889.146	64.4328	285.9813	6439.716	427.9455	0.0063
	2100	525.5172	567.5508	0.0666	5620.201	57.4335	310.8798	6698.64	426.9321	0.0081
E_n_F	2030	895.3479	594.153	0.0045	6856.259	97.4223	218.6946	5008.16	537.1632	0.0252
	2060	884.5947	664.0623	0.0549	6622.25	90.7065	259.6653	4898.259	787.6035	0.0324
	2080	876.9708	703.7667	0.0486	6477.269	86.8914	285.8301	4912.789	863.6364	0.027
	2100	869.0535	738.5724	0.0378	6340.155	83.529	311.1579	4919.229	945.4563	0.0378
E_s_F	2030	824.0976	594.837	0.072	7054.171	96.5232	218.7585	5008.352	410.3919	0.0252
	2060	858.5208	631.2915	0.0045	7100.912	89.0739	260.3349	4873.786	393.2649	0.0405
	2080	864.6057	659.8467	0.0045	7133.263	85.0986	287.2701	4786.09	391.0329	0.0171
	2100	868.2003	702.279	0.0045	7166.38	81.7866	313.6788	4699.972	374.9094	0.0189
SD	2030	887.7807	595.9134	0.0756	7075.222	99.7857	203.5728	5009.14	335.7234	0.0153
	2060	885.5001	680.805	0.0459	7146.095	95.697	205.155	4875.688	318.2283	0.0144
	2080	896.7078	729.0144	0.0045	7190.096	93.123	202.4442	4788.691	307.1349	0.0135
	2100	895.4784	789.201	0.0045	7231.588	90.6633	200.5695	4703.239	296.4726	0.0126

### 3.2 Quantification of the supply and demand of ecosystem services

This study calculated the *ES*_*r*_ of Urumqi in 1990, 1995, 2000, 2005, 2010, 2015, and 2020 via equations (2.4), (2.5), and (2.6), the results were shown in Table 10. The *ES*_*r*_ of Urumqi decreased in recent years and it was in a surplus condition, which meant the supply of ecosystem services in Urumqi satisfied the demand for ecosystem services, however, the degree of satisfaction was in decreasing trend. A widening gap between the supply and demand of ecosystem services will lead to a deterioration in ecosystem health.

**Table 10:** The ${ES}_{r}$ of Urumqi in recent years.

Year	1990	1995	2000	2005	2010	2015	2020
Farmland	−304.234	−289.64	−269.498	−281.596	−270.601	−228.785	−201.588
Forest	16665.18	17484.24	17992.84	18904.16	19648.76	20174.95	20667.89
Shrub	0.9405	1.85625	0.1485	0.1485	0.12375	0.12375	2.178
Grassland	105946.6	106848.6	108126.8	104065	101855.6	102032.1	99215.01
Water area	1660.914	1851.858	2140.569	2401.786	2618.09	2352.008	2436.303
Snow and ice	3709.843	4450.043	3973.952	3749.439	3992.398	4576.333	4295.309
Bare land	45476.79	44260.91	43591.88	45175.18	45901.09	46324.16	48435.55
Impervious surface	−3251.35	−4618.94	−5852.48	−6807.15	−8456.01	−9631.53	−10768.6
Wetland	0.18135	0.09765	0.0279	0.04185	0.0558	0.1953	0.2511
Total ${ES}_{r}$	169904.9	169989	169704.2	167207	165289.5	165599.5	164082.3

The *ES*_*r*_ of future scenarios were calculated via equations (2.1), (2.5), and (2.6) as well. The results were shown in Table 11. It can be seen that in the scenario of SD, the *ES*_*r*_ will be higher than that in other scenarios. This indicates that to ensure ecosystem stability and security, future development is more inclined to prioritize ecological considerations.

**Table 11:** The *ES*_*r*_ of Urumqi in future scenarios.

	2030	2060	2080	2100
BS	164144.5	161949.5	161126.4	159902.4
E_n_F	159872.1	152642.7	150723.1	148520
E_s_F	165850.6	167731.6	168967.1	171077.7
SD	167761.7	171032.3	172784.6	174965.4

### 3.3 Determination of the threshold of the supply and demand of ecosystem services

#### 3.3.1 Quantification of *R*_*SD*_ and *C*_*SD*_.

This study quantified the *R*_*SD*_ and *C*_*SD*_ of Urumqi via equation (2.2) and equation (2.3) in Section 2.3. The results were shown in Table 12 and Table 13. It can be seen that in recent years and regardless of the development scenario chosen in the future, the *R*_*SD*_ and *C*_*SD*_ both satisfy the requirements listed in equation (2.4).

**Table 12:** The *R*_*SD*_ and *C*_*SD*_ of Urumqi in recent years.

	1990	1995	2000	2005	2010	2015	2020
***R***_***SD***_	2.95	2.92	2.90	2.87	2.84	2.88	2.89
***C***_***SD***_	0.87	0.87	0.87	0.88	0.88	0.88	0.87

**Table 13:** The *R*_*SD*_ and *C*_*SD*_ of Urumqi in future scenarios.

Future scenarios	Indicators	2030	2060	2080	2100
BS	*R*_*SD*_	2.93	3.05	3.15	3.20
	*C*_*SD*_	0.87	0.86	0.86	0.85
E_n_F	*R*_*SD*_	2.69	2.51	2.48	2.43
	*C*_*SD*_	0.89	0.90	0.91	0.91
E_s_F	*R*_*SD*_	2.84	2.84	2.85	2.87
	*C*_*SD*_	0.88	0.88	0.88	0.88
SD	*R*_*SD*_	2.87	2.91	2.92	2.94
	*C*_*SD*_	0.88	0.87	0.87	0.87

#### 3.3.2 *Tr*_*SD*_ determination of Urumqi.

This study took use of inflection point analysis of the supply and demand of ecosystem services to set *Tr*_*SD*_. To obtain the inflection points, Origin 2023 was used to conduct an inflection point analysis on *ES*_*r*_. The inflection points were obtained via equation (2.7), and the *Tr*_*SD*_ was limited by equation (2.4).

The authors used “Origin” to calculate and analyze the inflection points of ${ES}_{r}$. The specific operation of how to achieve inflection points in Origin are as follows. First, input all data into the sheet, then choose “Analysis” tool, and then start “mathematics” to “differentiate” the data with different derivative orders.

**Fig 6:**
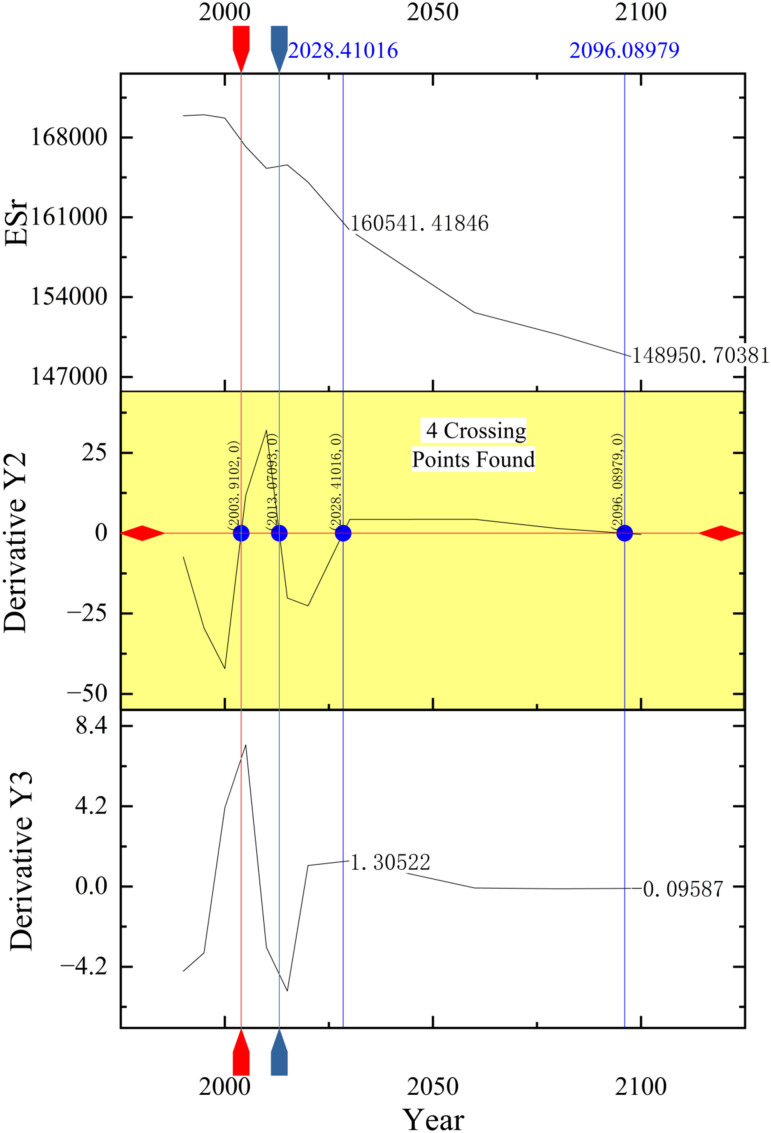
The inflection points of *ES*_*r*_ in the scenario of E_n_F.

Taking the inflection point analysis of *ES*_*r*_ in the scenario of E_n_F as an example, the differential calculus of *ES*_*r*_ was shown in Fig 6. Taking use of the level crossing tool in Origin, the red horizontal line in Fig 6 indicates that the second derivative is 0. The four vertical lines and the third derivative intersect can read the third-order derivative value, and in the case of the point with the second derivative of 0 changing around the plus and minus signs (equation 2.7), the intersection points of the vertical lines and the *ES*_*r*_ function curve were the inflection points of *ES*_*r*_ in the scenario of E_n_F. In the same way, other inflection points of *ES*_*r*_ in other scenarios were analyzed.

The points marked in “Derivative Y2” are the inflection points of *ES*_*r*_ in the scenario of E_n_F.

All inflection points of *ES*_*r*_ in Urumqi were shown in Table 14. The ordinates of inflection points were ranked in a positive direction, and the minimum value was substituted into equation (2.4) to confirm that it met the limitations of *Tr*_*SD*_. As previously mentioned, the *Tr*_*SD*_ represents a critical value for maintaining the balance between the supply and demand of ecosystem services. The results presented earlier demonstrate that the *ES*_*r*_ performs relatively well under the SD scenario. In line with the principle of ecological priority adopted in this study, the most ecologically favorable inflection point was selected as the *Tr*_*SD*_. That is, the *Tr*_*SD*_ in Urumqi was determined to be 148950.70. When *ES*_*r*_ was lower than 148950.70, it is necessary to adjust the constraints of regional economic and social activities to improve the supply capacity or demand of regional ecosystem services.

**Table 14:** The ordinates of the inflection points of *ES*_*r*_ in Urumqi.

Scenarios	BS	E_n_F	E_s_F	SD
**1**	(2003.91, 167751.30)	(2003.91, 167751.30)	(2003.91, 167751.30)	(2003.91, 167751.30)
**2**	(2058.69, 162045.47)	(2013.07, 165479.92)	(2058.53, 167639.17)	(2053.20, 170291.41)
**3**		(2028.41, 160541.42)	(2065.62, 168079.05)	(2077.94, 172604.31)
**4**		(2096.09, 148950.70)		

## 4 Discussions

This section contains three parts: 1) Discussions on the innovation and rationality of *Tr*_*SD*_ definition according to the definition itself; 2) Discussions on the feasibility of *Tr*_*SD*_ determination according to the methods themselves and the results of the case study; 3) Discussions on the reasonability of inflection point analysis according to the definition of *Tr*_*SD*_ and the result of inflection point analysis in the case study; and 4) Future work.

### 4.1 The innovation and rationality of *Tr*_*SD*_ definition

The definition of *Tr*_*SD*_ proposed in this study is innovative and rational. It can be regarded as a supplement to the concept of ecological threshold in terms of *ES*_*r*_.

In the “introduction” part, this study claimed that there was no definition of the threshold related to the supply and demand of ecosystem services based on the relationship between supply and demand. This study defined it, which was innovative.

According to Section 2.3, *Tr*_*SD*_ refers to the state in which the difference between the supply and demand of ecosystem services arrives at a tipping point. It set limitations on the relationship between the supply and demand of ecosystem services, containing the identification of *R*_*SD*_, *C*_*SD*_, and *ES*_*r*_, highlighting the difference between the supply of ecosystem services and the demand for ecosystem services. When the state of the supply and demand of ecosystem services arrives at its tipping point, the state of the ecosystem changes suddenly, and the *Tr*_*SD*_ is generated. This study defined *Tr*_*SD*_ based on the changes in the supply and demand of ecosystem services and is rational theoretically.

### 4.2 The feasibility of *Tr*_*SD*_ determination methods

According to Section 2.4, this study proposed a systematic method for *Tr*_*SD*_ determination, including the GeoSOS-FLUS model (future land use prediction), LULC matrix model (*ES*_*r*_ quantification), and infection point analysis (*ES*_*r*_ analysis and *Tr*_*SD*_ determination). Theoretically, the methods introduced in Section 2.4 are feasible.

To verify the feasibility of the above methods practically, this study took Urumqi as the case city and determined *Tr*_*SD*_ via the above methods. The details are as follows.

(1) This study predicted future land use in different scenarios via the GeoSOS-FLUS model. The results were shown in Section 3.1 (Table 9). Among them, the land use in the scenario of BS was consistent with the characteristics of land use change shown in recent years. It can be seen that the GeoSOS-FLUS model is feasible to predict future land use.
(2) This study quantified *ES*_*r*_ via modified LULC matrix. Since the land use types are different from Burkhard’s researches [17,18,30], this study modified the intensities of the LULC matrix in Section 2.4.2. This study made a comparison between the results generated according to the intensities used by Wu et al. [32] and the results generated according to the modified intensities in this study to quantify the supply and demand of ecosystem services in Urumqi in the same period. From the perspective of the overall trend of *ES*_*r*_, the two presented similar trends, indicating that the LULC matrix model modified in this study was feasible.
(3) This study determined *Tr*_*SD*_ via the analysis of the tipping points found by inflection point analysis of *ES*_*r*_. *Tr*_*SD*_ determination aims to maintain the continuous surplus of the supply and demand of ecosystem services and to promote eco-friendly development. According to the definition of *Tr*_*SD*_, the determination of *Tr*_*SD*_ is to find the tipping point of *ES*_*r*_ for maintaining the matching and basic coordination of the supply and demand of ecosystem services. So, the minimum ordinate of the inflection points listed in Table 14 which meet the requirements listed in equation (2.3) was chosen to be the *Tr*_*SD*_ of Urumqi. It can be seen that the *Tr*_*SD*_ determined by the inflection point analysis is feasible.

### 4.3 The reasonableness of inflection point analysis used for *Tr*_*SD*_ determination

The inflection point analysis can analyze the changes in *ES*_*r*_ and *Tr*_*SD*_ determination. It is reasonable to be used in *Tr*_*SD*_ determination.

Inflection point analysis is often used in the field of mathematics [37], economics and financial management [38], etc. In this study, the inflection point analysis method was used to analyze *ES*_*r*_ in Urumqi for *Tr*_*SD*_ determination, expanding the application of inflection point analysis in the field of ecology and ecosystem services.

According to the definition of *Tr*_*SD*_, the determination of *Tr*_*SD*_ is to find the tipping point of the changing *ES*_*r*_. This study took use of inflection points to represent the tipping points, satisfying the mathematical meaning of inflection points. This study took Urumqi as the case city and determined the *Tr*_*SD*_ of Urumqi via inflection point analysis in section 3.3.2. The determined *Tr*_*SD*_ in Urumqi was the minimum ordinate of the inflection points listed in Table 14 and has been verified to meet the requirements listed in equation (2.3), indicating that the inflection points analysis of *ES*_*r*_ is reasonable regarding *Tr*_*SD*_ determination.

### 4.4 Future work

As mentioned at the beginning, the current lack of research on the threshold of ecosystem service supply and demand relationship is a gap in ecological conservation. This study proposes the concept of *Tr*_*SD*_ and a method for its determination, which can serve as a reference for planners and policymakers in daily decision-making processes related to industrial and commercial land use. Although we have demonstrated the feasibility and rationality of the proposed method, a series of supplementary studies will be necessary in the future.

(1) Uncertainties of future scenarios and land use

The series of methods adopted in this study were derived through comparative analysis and model simulation. Additionally, the analysis of future land use relies on different predefined scenarios, both of which involve certain degrees of uncertainty. In future research, we plan to incorporate studies of past years to better understand the logic of land use changes, while also integrating socioeconomic and other relevant factors to gradually improve the accuracy of the simulations.

(2) Limits on the set of *Tr*_*SD*_

This study primarily employs inflection point analysis to determine the *Tr*_*SD*_. This process involves two main sources of uncertainty: first, the uncertainty associated with future land use, as mentioned earlier; and second, the presence of multiple inflection points identified during the analysis. In this study, the SD scenario was selected, adhering to the principle of ecological priority. However, in real-world social contexts, numerous additional factors must be considered. Therefore, to determine the *Tr*_*SD*_ in practical applications, it is essential to further compare the threshold values derived from different scenarios. This comparison will enable the selection of an inflection point that is better aligned with actual socioeconomic conditions as the final threshold.

(3) Lack of more typical cases

Additionally, this study focused solely on Urumqi as a case study. In reality, more case studies are needed to validate the adaptability of the research method proposed in this paper.

Urumqi is a typical semi-arid region. In subsequent research, comparative studies could be conducted by selecting different types of areas and cities with varying economic strengths as case studies. In addition to analyzing the selection of different scenario models, the universality of the method proposed in this study could be further examined. For example, representative Chinese cities such as Beijing and Shanghai could be considered as case studies for further validation.

(4) Further deliberation

This study introduces the concept of *Tr*_*SD*_, which not only addresses a gap in existing research but also provides a reference for policymakers and urban planning authorities. Currently, climate change, ecological security and conservation are critical global issues. The *Tr*_*SD*_ can reflect the security of ecosystem services, and demonstrate the balance between human activities and ecological systems. In urban planning, where land use types and surrounding infrastructure must be clearly defined, this study offers a distinct advantage: it helps maximize economic and social value while ensuring ecological security and maintaining the balance of ecosystem services.

In future research and practical applications, the proposed method can be compared with different ecosystem service valuation approaches. Additionally, integrating the *Tr*_*SD*_ into existing ecosystem service assessment frameworks should be considered. Furthermore, it could serve as a validation tool for delineating ecological protection redlines, ensuring ecological security within these designated areas.

## 5 Conclusions

At present, the concept of *Tr*_*SD*_ has not been studied, but it is of great significance in maintaining the continuous surplus of the supply and demand of ecosystem services and promoting eco-friendly development. This study aims to definite *Tr*_*SD*_ and propose a systematic method for *Tr*_*SD*_ determination. According to the results of the case study and the discussions on the definition of *Tr*_*SD*_ and the determination method for *Tr*_*SD*_, this study came out with the following conclusions:

a) The threshlod of the supply and demand of ecosystem services (*Tr*_*SD*_) was defined. The proposed systematic method of *Tr*_*SD*_ determination includes future land use prediction via GeoSOS-FLUS, *ES*_*r*_ evaluation via the LULC matrix model (the modified intensities of the LULC matrix are rational), and inflection point analysis of *ES*_*r*_.
b) The *Tr*_*SD*_ proposed in this study can serve as a reference standard for urban planning and development. For instance, during land use planning, it can be used as an indicator for ecological conservation to test the balance between ecological and socio-economic considerations. Furthermore, in the future, *Tr*_*SD*_ could be utilized as one of the validation indicators for delineating ecological protection redlines. It could also be integrated with ecosystem service valuation methods for further optimization.

## Supporting information

S1 File Supporting information. data resources availability. (DOCX)

S2 File Supporting information-appendixes A to C. (ZIP)

## References

[1] Millennium Ecosystem Assessment editor. Ecosystems and human well-being: synthesis. Washington, DC: Island Press; 2005.

[2] Sasaki T, Furukawa T, Iwasaki Y, Seto M, Mori AS. Perspectives for ecosystem management based on ecosystem resilience and ecological thresholds against multiple and stochastic disturbances. Ecol Indic. 2015;57:395–408.

[3] Standish RJ, Hobbs RJ, Mayfield MM, Bestelmeyer BT, Suding KN, Battaglia LL, et al. Resilience in ecology: abstraction, distraction, or where the action is?. Biol Conserv. 2014;177:43–51. doi: 10.1016/j.biocon.2014.06.008

[4] Qian SS. Ecological threshold and environmental management: a note on statistical methods for detecting thresholds. Ecol Indic. 2014;38:192–7.

[5] Wang S, Wei Y. Overview and prospects for ecological safety threshold research. Acta Prataculturae Sinica. 2017;26(1):195–205.

[6] Xu H, Tian H. The theory of ecological threshold and its application on biodiversity conservation. Inner Mongolia Sci Technol Econ. 2014;(7):49–50.

[7] Yu G-R, Zhang X-M, Zhao D-S, Deng S-Q. Discussion on the scientific concepts of regional resources and environmental carrying capacity and its ecological basis. Ying Yong Sheng Tai Xue Bao. 2022;33(3):577–90. doi: 10.13287/j.1001-9332.202203.020 35524508

[8] Rockström J, Steffen W, Noone K, Persson A, Chapin FS 3rd, Lambin EF, et al. A safe operating space for humanity. Nature. 2009;461(7263):472–5. doi: 10.1038/461472a 19779433

[9] Zhao D-S, Zhang X-M, Deng S-Q, Yu G-R. Evaluation theory and method of regional resources and environmental carrying capacity. Ying Yong Sheng Tai Xue Bao. 2022;33(3):591–602. doi: 10.13287/j.1001-9332.202203.024 35524509

[10] Hai-Ping T, Jiao C, Xue Hai-Li A. Ecological thresholds: Concept, methods and research outlooks. Chin J Plant Ecol. 2015;39(9):932–40.

[11] Xu X-L, Yu G-R. Theories of ecosystem vulnerability, adaptability and catastrophe based on the mechanisms of ecological succession. Ying Yong Sheng Tai Xue Bao. 2022;33(3):623–8. doi: 10.13287/j.1001-9332.202203.025 35524512

[12] Liang X, Liu X, Li X, Chen Y, Tian H, Yao Y. Delineating multi-scenario urban growth boundaries with a CA-based FLUS model and morphological method. Landscape Urban Plan. 2018;177:47–63. doi: 10.1016/j.landurbplan.2018.04.016

[13] Nie W, Xu B, Yang F, Shi Y, Liu B, Wu R, et al. Simulating future land use by coupling ecological security patterns and multiple scenarios. Sci Total Environ. 2023;859(Pt 1):160262. doi: 10.1016/j.scitotenv.2022.160262 36400298

[14] Li Z. Response of ecosystem service to climate and land use and scenario simulation in Beijing. Beijing Forestry University; 2021. https://link.cnki.net/doi/10.26949/d.cnki.gblyu.2021.000808

[15] Wang Q, Guan Q, Sun Y, Du Q, Xiao X, Luo H, et al. Simulation of future land use/cover change (LUCC) in typical watersheds of arid regions under multiple scenarios. J Environ Manage. 2023;335:117543. doi: 10.1016/j.jenvman.2023.117543 36848808

[16] Liu X, Liang X, Li X, Xu X, Ou J, Chen Y, et al. A future land use simulation model (FLUS) for simulating multiple land use scenarios by coupling human and natural effects. Landscape Urban Plan. 2017;168:94–116. doi: 10.1016/j.landurbplan.2017.09.019

[17] Burkhard B, Kroll F, Müller F, Windhorst W. Landscapes’ capacities to provide ecosystem services - A concept for land-cover based assessments. LO. 2009;15:1–22. doi: 10.3097/lo.200915

[18] Burkhard B, Kroll F, Nedkov S, Müller F. Mapping ecosystem service supply, demand and budgets. Ecol Indic. 2012;21:17–29.

[19] Huimin L. The impact of human behavior on ecological threshold: Positive or negative?—Grey relational analysis of ecological footprint, energy consumption and environmental protection. Energy Policy. 2013;56:711–9. doi: 10.1016/j.enpol.2013.01.044

[20] Sasaki T, Koyama A, Okuro T. Coupling structural and functional thresholds for vegetation changes on a mongolian shrubland. Ecol Indic. 2018;93:1264–75.

[21] Stagg CL, Osland MJ, Moon JA, Hall CT, Feher LC, Jones WR. Quantifying hydrologic controls on local- and landscape-scale indicators of coastal wetland loss. Ann Bot. 2019. doi: mcz144 10.1093/aob/mcz144 PMC7442328 31532484

[22] Sasaki T, Okayasu T, Jamsran U, Takeuchi K. Threshold changes in vegetation along a grazing gradient in Mongolian rangelands. J Ecol. 2008;96(1):145–54.

[23] Shi X, Chen H, Shi W, Wang L, Pan P, Liang Y. Risk assessment of ecosystems production based on the thresholds identification: the case study of farming-pastoral ecotone in Northern China. Ecol Environ Sci. 2017;26(1):6–12.

[24] Sun R, Jin X, Han B, Liang X, Zhang X, Zhou Y. Does scale matter? Analysis and measurement of ecosystem service supply and demand status based on ecological unit. Environ Impact Assess Rev. 2022;95:106785. doi: 10.1016/j.eiar.2022.106785

[25] Guan Q, Hao J, Ren G, Li M, Chen A, Duan W, et al. Ecological indexes for the analysis of the spatial–temporal characteristics of ecosystem service supply and demand: a case study of the major grain-producing regions in Quzhou, China. Ecol Indic. 2020;108:105748.

[26] Chen W, Chi G. Spatial mismatch of ecosystem service demands and supplies in China, 2000–2020. Environ Monit Assess. 2022;194(4):295. 35333991 10.1007/s10661-022-09981-y

[27] Deng H, Zhou X, Liao Z. Ecological redline delineation based on the supply and demand of ecosystem services. Land Use Policy. 2024;140:107109. doi: 10.1016/j.landusepol.2024.107109

[28] Liao Z. Quantitative judgement and classification system for coordinated development of environment and economy — A case study of the city group in the Pearl River Delta. Trop Geograph. 1999;(2):76–82.

[29] Sleeter BM, Sohl TL, Bouchard MA, Reker RR, Soulard CE, Acevedo W, et al. Scenarios of land use and land cover change in the conterminous United States: utilizing the special report on emission scenarios at ecoregional scales. Global Environ Change. 2012;22(4):896–914. doi: 10.1016/j.gloenvcha.2012.03.008

[30] Burkhard B, Müller A, Müller F, Grescho V, Anh Q, Arida G, et al. Land cover-based ecosystem service assessment of irrigated rice cropping systems in southeast asia—an explorative study. Ecosyst Serv. 2015;14:76–87.

[31] Tao Y, Wang H, Ou W, Guo J. A land-cover-based approach to assessing ecosystem services supply and demand dynamics in the rapidly urbanizing Yangtze River Delta region. Land Use Policy. 2018;72:250–8. doi: 10.1016/j.landusepol.2017.12.051

[32] Wu X, Liu S, Zhao S, Hou X, Xu J, Dong S, et al. Quantification and driving force analysis of ecosystem services supply, demand and balance in China. Sci Total Environ. 2019;652:1375–86. doi: 10.1016/j.scitotenv.2018.10.329 30586822

[33] Li W, Wang Y, Xie S, Cheng X. Spatiotemporal evolution scenarios and the coupling analysis of ecosystem health with land use change in southwest China. Ecol Eng. 2022;179:106607.

[34] Chen G, Li X, Liu X, Chen Y, Liang X, Leng J, et al. Global projections of future urban land expansion under shared socioeconomic pathways. Nat Commun. 2020;11(1):537. doi: 10.1038/s41467-020-14386-x 31988288 PMC6985221

[35] Zhang S, Liu X, Yan S, Zhan Q, Liu T. Delimitation of urban development boundaries using two basic evaluations and FLUS-UGB: A case study of Changchun. Trop Geograph. 2019;39(3):377–86.

[36] Pontius RG, Boersma W, Castella JC, Clarke K, De Nijs T, Dietzel C. Comparing the input, output, and validation maps for several models of land change. Ann Reg Sci. 2008;42(1):11–37.

[37] Bai L. The analysis of singularities and inflection points on plannar C-B spline curve. College Mathematics. 2010;26(6):102–6.

[38] Huang L. Analysis of China’s short-term market interest rate trend and inflection points. New Finance. 2017;10:25–31.

