# Supplementary material for: Research on the threshold of the supply and demand of ecosystem services
Source: PLoS One. 2026 Feb 2;21(2):e0339122. doi: 10.1371/journal.pone.0339122 (PMC12863479; doi:10.1371/journal.pone.0339122)
Supplement: S1 File — (DOCX) [file pone.0339122.s001.docx]

# Supporting information

1. **Remote sensing images of land monitoring**

The images and related data can be collected from Yang and Huang’s research[^[[1]](#endnote-0)^] (https://doi.org/10.5194/essd-13-3907-2021). we have added the footnote in the manuscript.

Also, the raw data can be downloaded from this website: <https://zenodo.org/records/12779975.> It is free to be downloaded.

1. **DEM, grids of road nets and water and GDP data, etc.**

DEM data can be downloaded from the website of Resource and Environmental Science Data Platform if you are the member of the platform. The SRTM 30m data (https://www.resdc.cn/data.aspx?DATAID=217) is not free to download. So as the grids of road and water. And we decided to not give this raw material to protect these data.

The GDP data was provided by Xu[^[[2]](#endnote-1)^]. It can be downloaded from this link: <https://www.resdc.cn/DOI/DOI.aspx?DOIID=33.>

1. **Other social and economic data**

As we have stated in the manuscript, the economic and other necessary data were collected from our national data platform and the statistical yearbook of the case city for the corresponding year. These data may be downloaded if you have the accessibility of the website. So, here we decided not to public with the manuscript to protect the data.

1. [] Yang, J. and Huang, X.: The 30 m annual land cover dataset and its dynamics in China from 1990 to 2019, Earth Syst. Sci. Data, 13, 3907–3925, https://doi.org/10.5194/essd-13-3907-2021, 2021. [↑](#endnote-ref-0)
2. [] Xu, X. China GDP Spatial Distribution Kilometer Grid Dataset. Resource and Environmental Science Data Registration and Publication System (http://www.resdc.cn/DOI),2017.DOI:10.12078/2017121102. [↑](#endnote-ref-1)
