# Supplementary material for: Research on the threshold of the supply and demand of ecosystem services
Source: PLoS One. 2026 Feb 2;21(2):e0339122. doi: 10.1371/journal.pone.0339122 (PMC12863479; doi:10.1371/journal.pone.0339122)
Supplement: S2 File — (ZIP) [file pone.0339122.s002.zip › Appendix A. the details of the modules of the GeoSOS-FLUS model.docx]

# Appendix A. GeoSOS-FLUS modules

This study took use of the GeoSOS-FLUS model to predict future land use in different scenarios. The model contains four modules.

a) ANN-based (artificial neural networks-based) probability-of-occurrence estimation. This part integrated economic, social, climate, and other driving factors or limiting factors and carried out the suitability probability simulation of various future land use changes.

b) Self-adaptive inertia and competition mechanism cellular automata (CA). This part took use of the historical land use map, the suitability probability map carried out in “ANN-based probability-of-occurrence estimation”, the demand for future land use, the land-use conversion cost matrix of different development scenarios, and neighborhood factor intensities to simulate future land use in different scenarios.

c) Markov prediction. This part was used to predict future land use demand for CA simulation.

d) Precision validation. This part contained the Kappa coefficient and FoM coefficient, which were used for the verification of the accuracy of future land use simulation.
