# Supplementary material for: Research on the threshold of the supply and demand of ecosystem services
Source: PLoS One. 2026 Feb 2;21(2):e0339122. doi: 10.1371/journal.pone.0339122 (PMC12863479; doi:10.1371/journal.pone.0339122)
Supplement: S2 File — (ZIP) [file pone.0339122.s002.zip › Appendix B. The details of the deficit operation to predict future lande use.docx]

# Appendix B. Steps of GeoSOS-FLUS’s operation

Step 1: Data prediction. Regarding land use prediction via GeoSOS-FLUS model, the following data shall be obtained: at least two periods of land use images, the images of the driving factors of land use change, the land-use conversion cost matrix of different development scenarios, and neighborhood factor intensities.

Step 2: The trial of land use prediction. Two periods of historical land use were chosen. One was regarded as the base period, and the other was regarded as the prediction period. Firstly, the “ANN-based probability-of-occurrence estimation” was carried out to obtain the suitability probability map. Then, the CA simulation was carried out to simulate and predict land use image of the prediction period.

Step 3: The verification of the accuracy of the prediction. The actual land use image of the prediction periods and the predicted land use image obtained by CA simulation were input into the Kappa coefficient and FoM coefficient modules to verify the accuracy of the model.

Step 4: Prediction of the demand for future land use. Under the premise of satisfying the accuracy verification, the Markov Chain module was used to predict the demand for future land use.

Step 5: Future land use prediction in different scenarios. Under the premise of satisfying the accuracy verification and obtaining future land use demand, the prediction period in "the trial of land use prediction" was regarded as the base period, and the year to be predicted was regarded as the forecast period. The “ANN-based probability-of-occurrence estimation” and CA simulation were repeated to predict future land use in different scenarios.
