# Supplementary material for: Research on the threshold of the supply and demand of ecosystem services
Source: PLoS One. 2026 Feb 2;21(2):e0339122. doi: 10.1371/journal.pone.0339122 (PMC12863479; doi:10.1371/journal.pone.0339122)
Supplement: S2 File — (ZIP) [file pone.0339122.s002.zip › Appendix C. the spatial distributions of future land use of Urumqi.docx]

# Appendix C. The spatial distributions of future land use


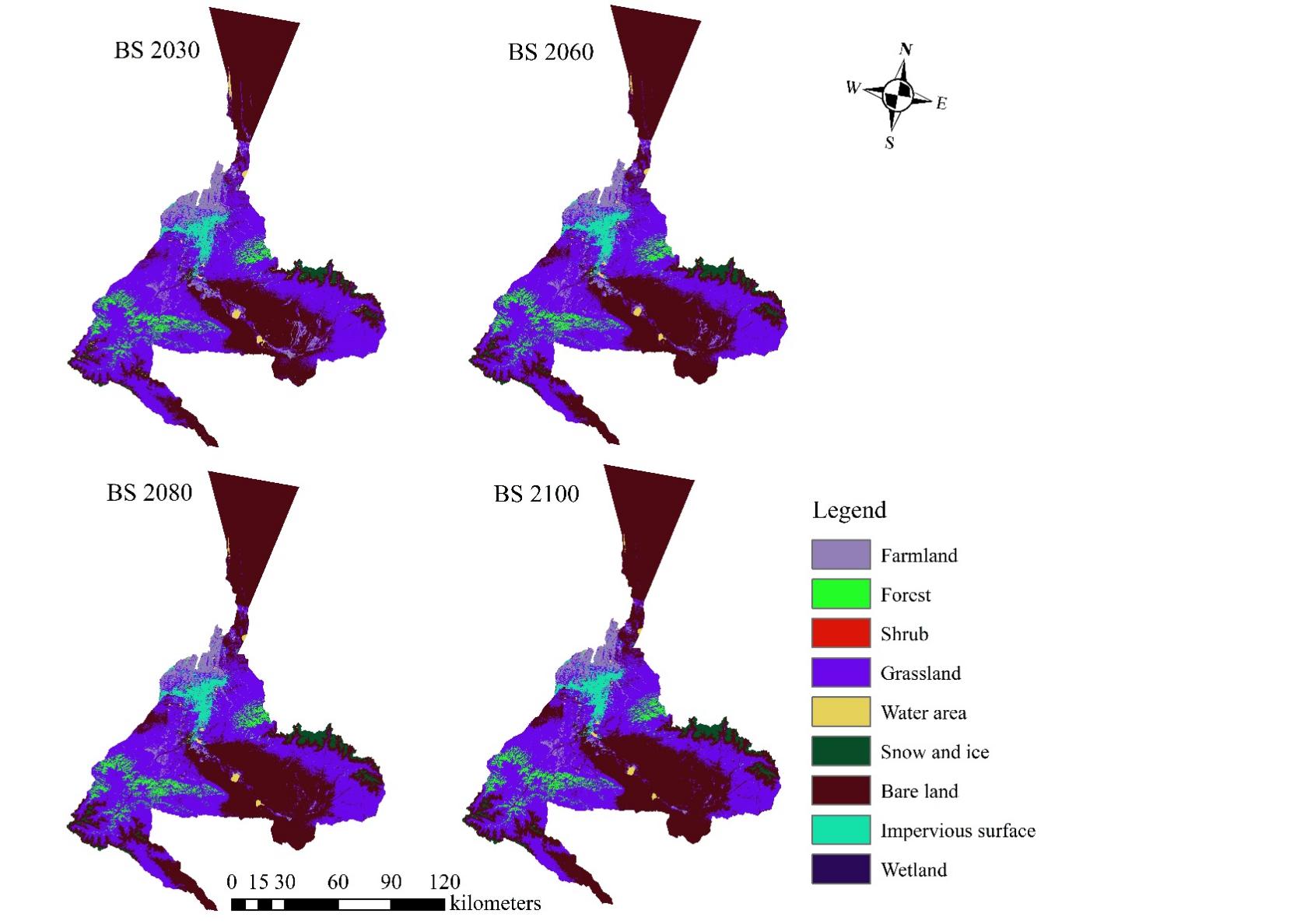


Fig C.1 Spatial distribution of land use (BS)


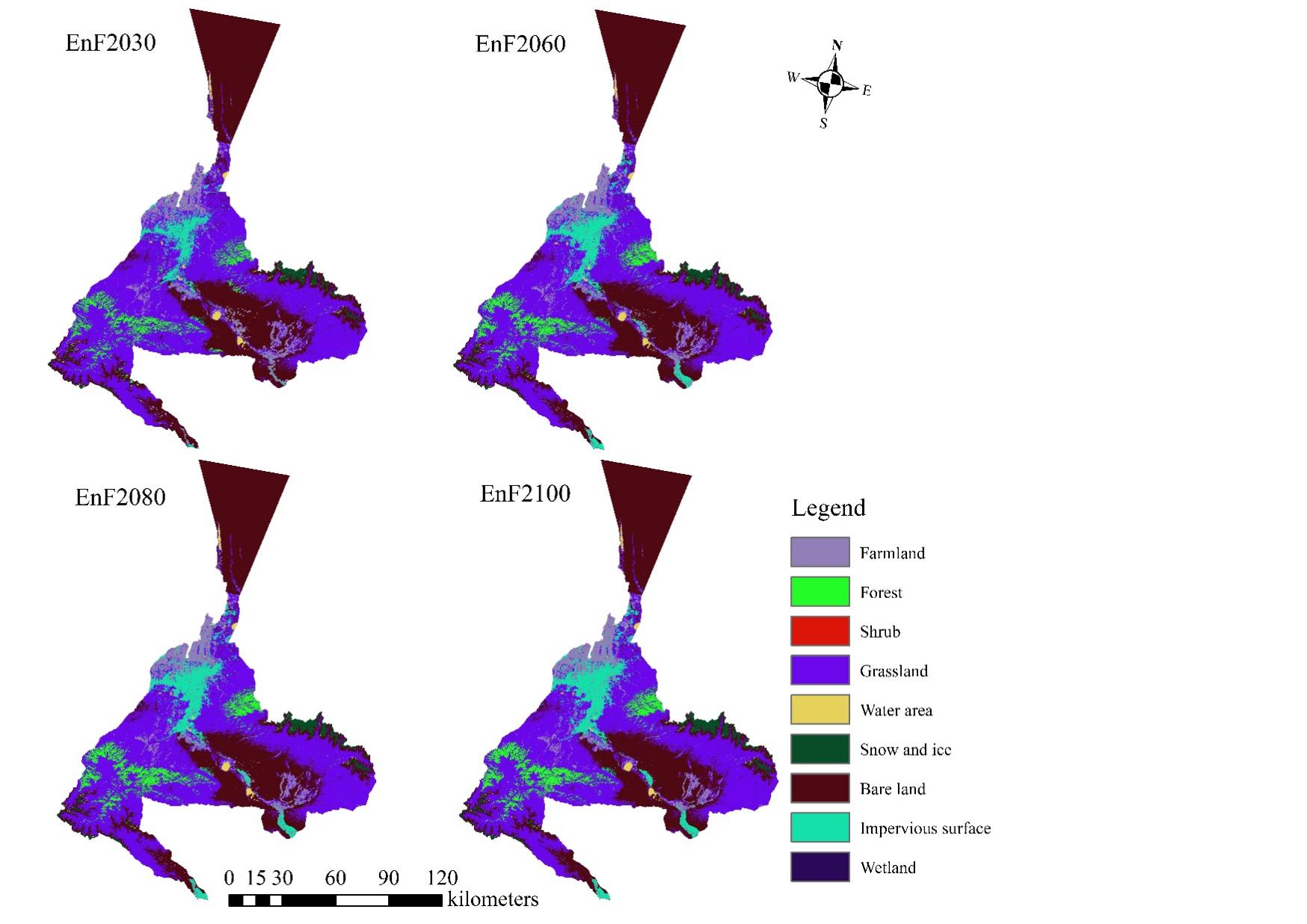


Fig C.2 Spatial distribution of land use (E_n_F)


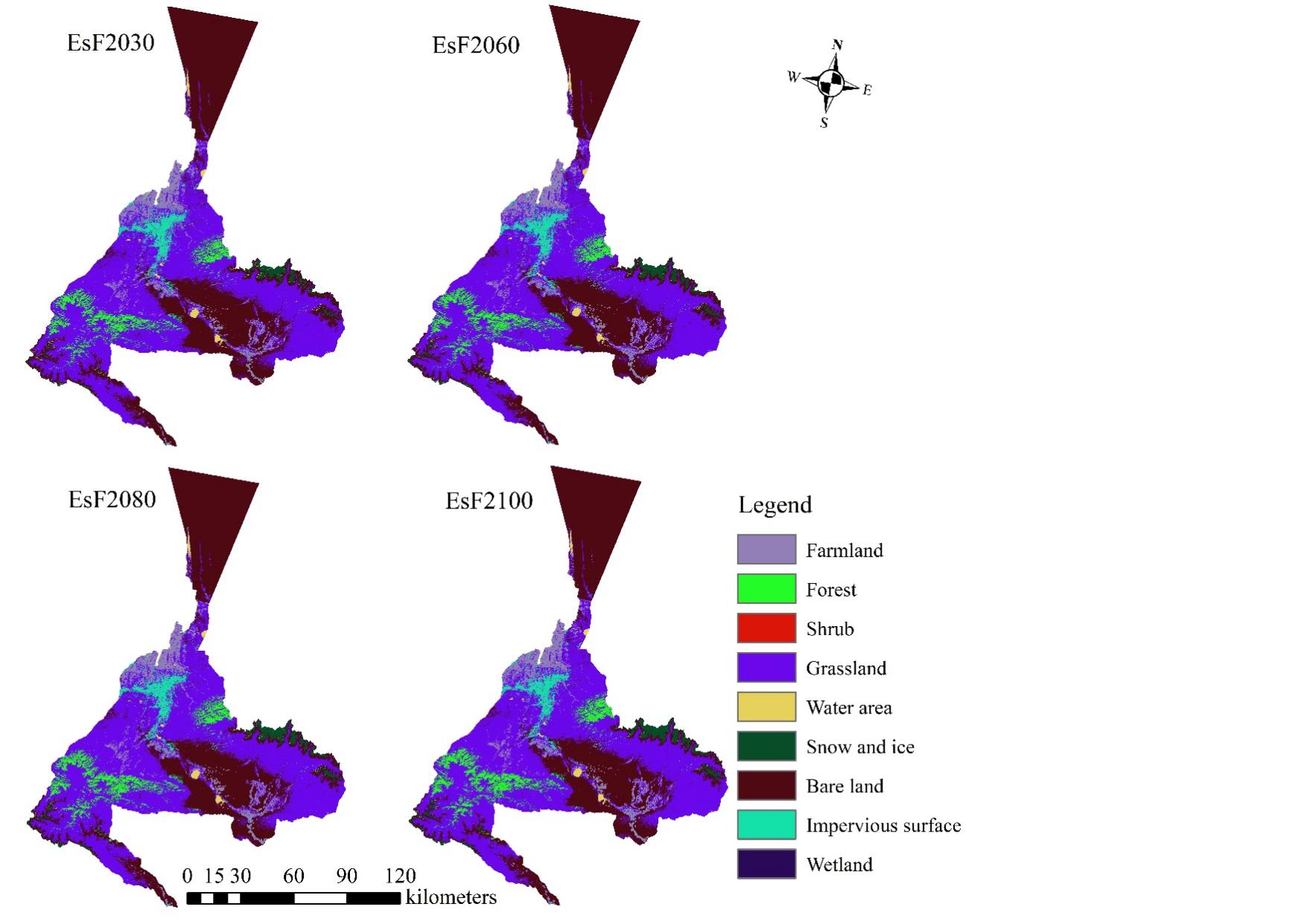


Fig C.3 Spatial distribution of land use (E_s_F)


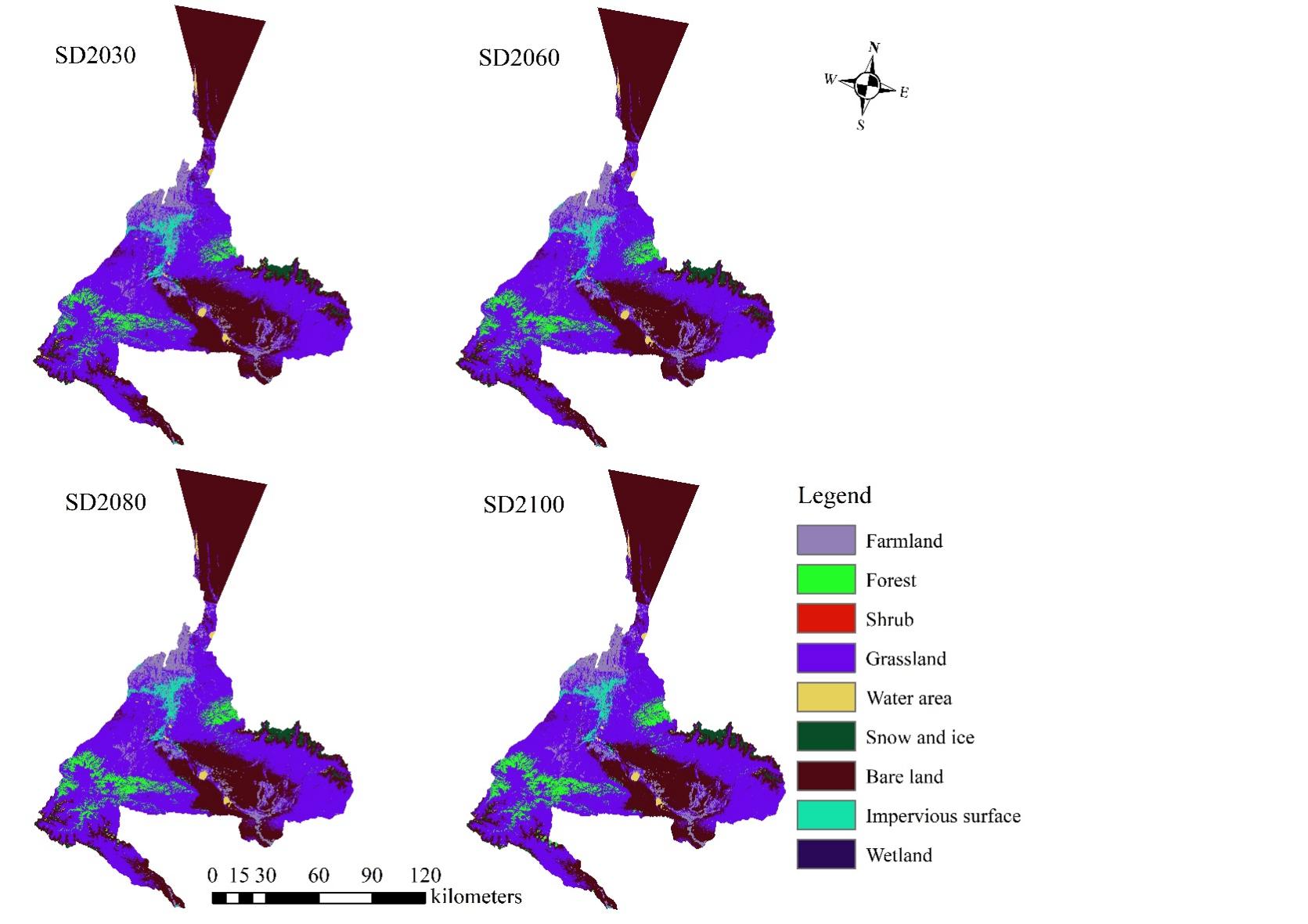


Fig C.4 Spatial distribution of land use (SD)
