# Supplementary figures and images for: Research on the threshold of the supply and demand of ecosystem services
Source: PLoS One. 2026 Feb 2;21(2):e0339122. doi: 10.1371/journal.pone.0339122 (PMC12863479; doi:10.1371/journal.pone.0339122)

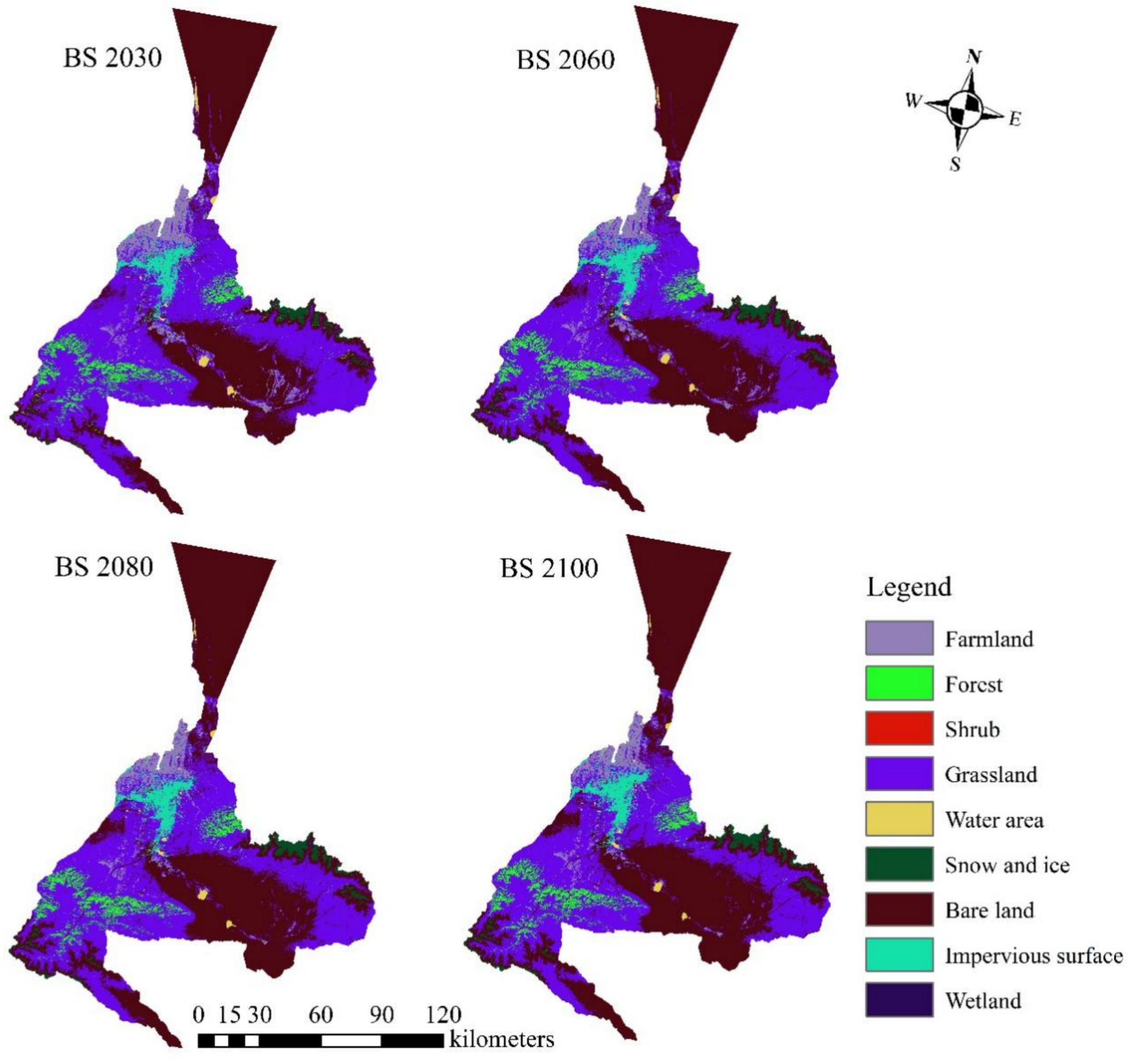

Supplement: S2 File — (ZIP) [file pone.0339122.s002.zip › Fig C.1 Spatial distribution of land use (BS).png]

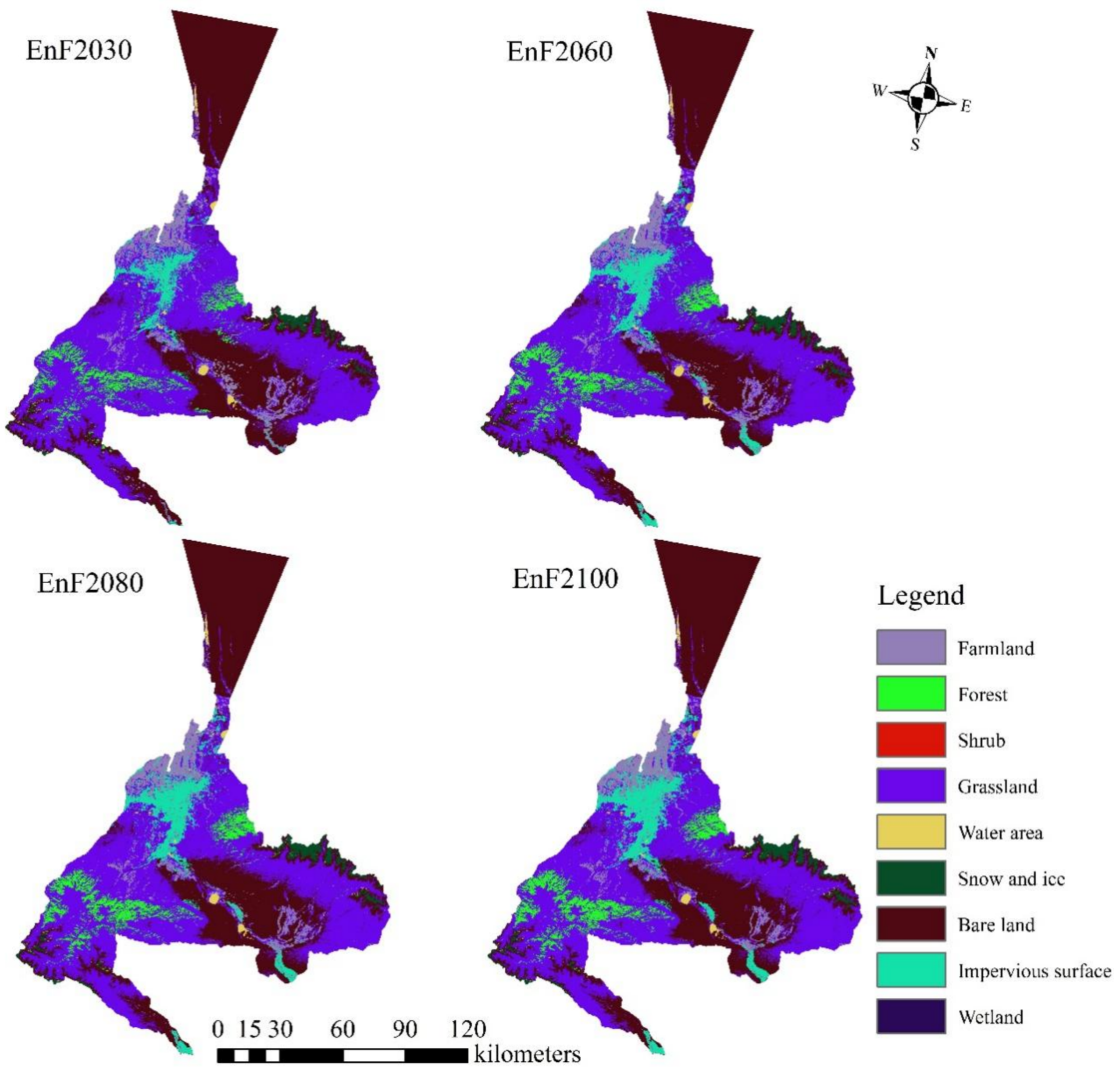

Supplement: S2 File — (ZIP) [file pone.0339122.s002.zip › Fig C.2 Spatial distribution of land use (EnF).png]

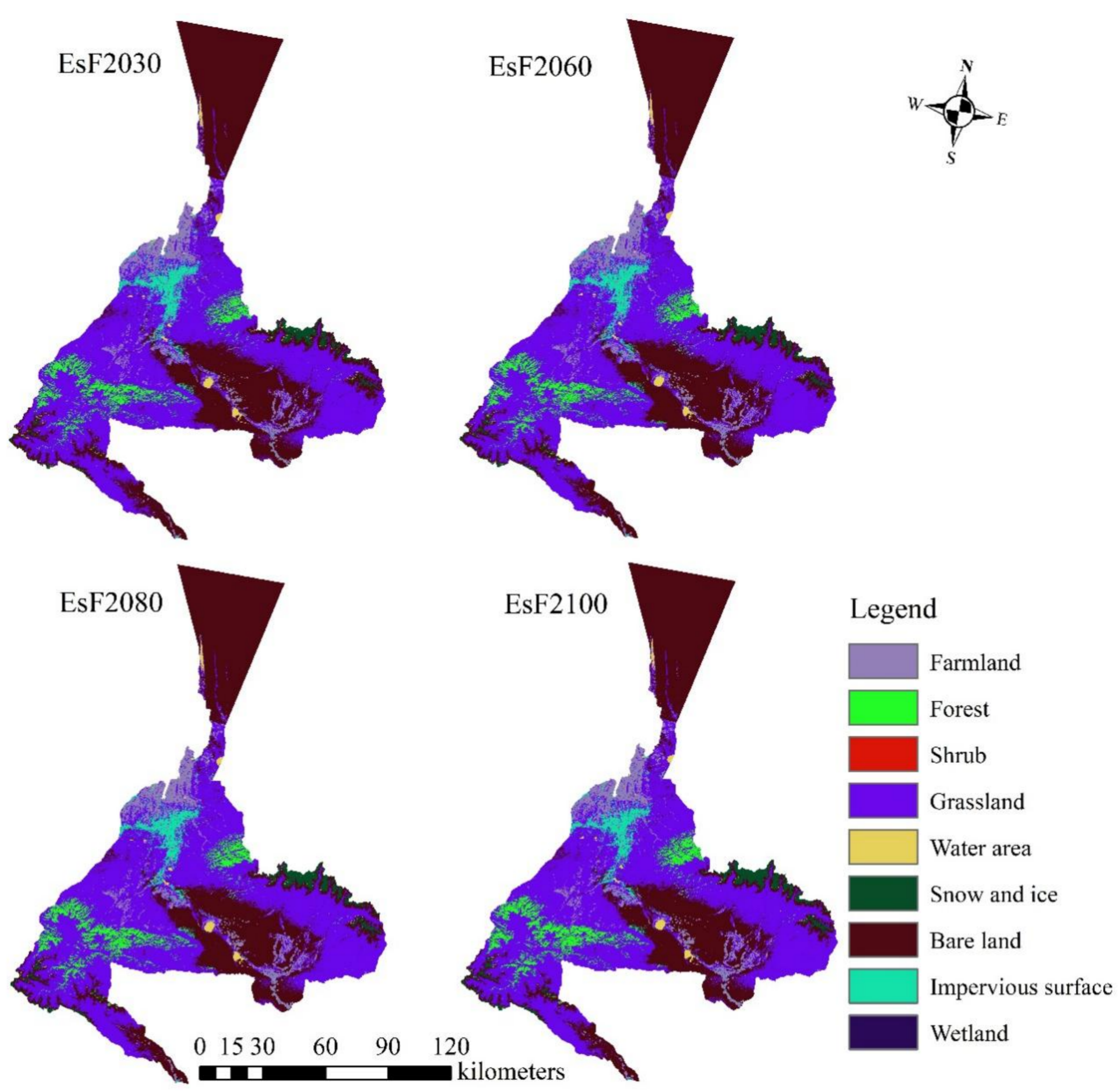

Supplement: S2 File — (ZIP) [file pone.0339122.s002.zip › Fig C.3 Spatial distribution of land use (EsF).png]

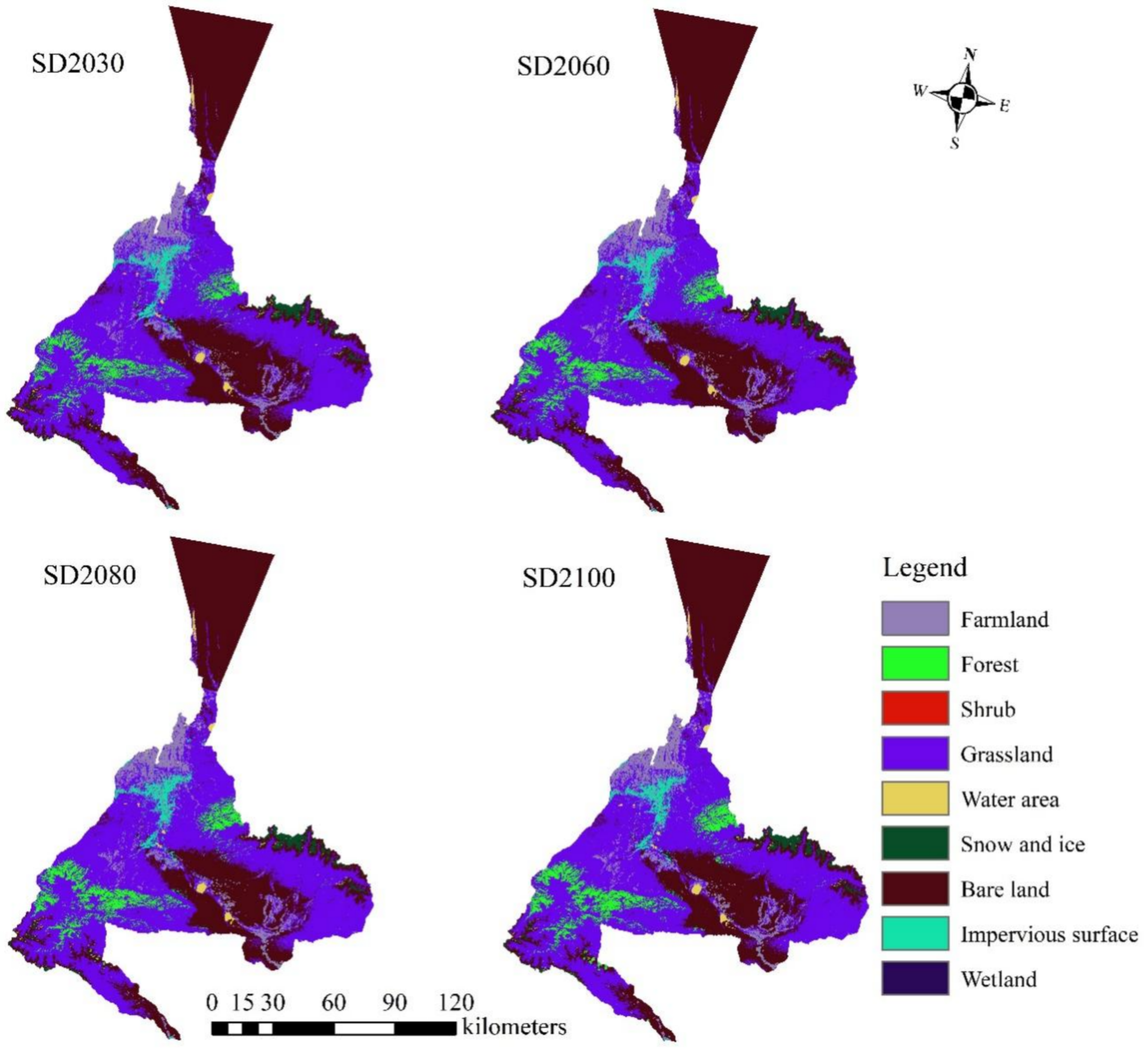

Supplement: S2 File — (ZIP) [file pone.0339122.s002.zip › Fig C.4 Spatial distribution of land use (SD).png]
